# Conditions under which Arousal Does and Does Not Elevate Height Estimates

**DOI:** 10.1371/journal.pone.0092024

**Published:** 2014-04-03

**Authors:** Justin Storbeck, Jeanine K. Stefanucci

**Affiliations:** 1 Department of Psychology, Queens College, City University of New York, Flushing, New York, United States of America; 2 Department of Psychology, University of Utah, Salt Lake City, Utah, United States of America; Birkbeck, University of London, United Kingdom

## Abstract

We present a series of experiments that explore the boundary conditions for how emotional arousal influences height estimates. Four experiments are presented, which investigated the influence of context, situation-relevance, intensity, and attribution of arousal on height estimates. In Experiment 1, we manipulated the environmental context to signal either danger (viewing a height from above) or safety (viewing a height from below). High arousal only increased height estimates made from above. In Experiment 2, two arousal inductions were used that contained either 1) height-relevant arousing images or 2) height-irrelevant arousing images. Regardless of theme, arousal increased height estimates compared to a neutral group. In Experiment 3, arousal intensity was manipulated by inserting an intermediate or long delay between the induction and height estimates. A brief, but not a long, delay from the arousal induction served to increase height estimates. In Experiment 4, an attribution manipulation was included, and those participants who were made aware of the source of their arousal reduced their height estimates compared to participants who received no attribution instructions. Thus, arousal that is attributed to its true source is discounted from feelings elicited by the height, thereby reducing height estimates. Overall, we suggest that misattributed, embodied arousal is used as a cue when estimating heights from above that can lead to overestimation.

## Introduction

A growing body of work suggests that emotions influence perceptual judgments. For example, threatening objects may appear closer than neutral objects [1], [2], sadness can make hills appear steeper [3], fearful faces may increase the ability to see contrast [4], and heights may appear taller when afraid [5], [6]. Emotions are typically described as having multiple components, which include arousal/activation and motivation/valence [7]–[9]. In its simplest form, the motivation or valence component invokes a goal to either approach (good) or withdraw from (bad) objects or situations [8], [10], [11]. For instance, people will withdraw from a dangerous snake and approach a cute cuddly baby. In contrast, the arousal/activation component signals the level of activation of the motivational system [12] and the consciously perceived urgency of the situation [13]. Russell and Barrett [12] suggest that arousal reflects changes in the sympathetic nervous system, the autonomic nervous system, or the endocrine system. For example, snakes may invoke a withdrawal motivation, but arousal signals the immediacy of action, which can be dependent on the type of snake. A King Cobra requires immediate withdrawal and the activation of the physiological system, whereas a garden snake may not. Within the context of height, people are often motivated to avoid a height, but does arousal influence the urgency to withdraw?

In the case of perception, increased arousal may alter representations of the environment by the intensification of feelings [e.g.], [ of danger or anger, see 13, 14]. This intensification could be taken into account when estimating the spatial layout of an environment, especially in situations where visual cues specifying layout (such as the horizon) may be ambiguous (e.g., when standing on a balcony or the top of a hill). If arousal biases perceptual judgments of spatial layout, then this could also contribute to both a feed-forward and feed-back loop, in which increased arousal biases perceptual judgments, which then leads to further arousal, and so on. We argued that such a mechanism could have led to the height overestimation observed in our previous work [6]. Across four experiments, we showed that arousal induced by viewing height-relevant arousing pictures increased estimates of a height that was viewed from above. These findings suggest that arousal is a key component of emotion that is utilized when estimating heights.

However, the motivated perception account suggests that arousal does not influence the perceived proximity to objects [see 15 for a review]. This account suggests that objects that invoke desirability or threat require action resulting in changes in the perceived proximity to those objects. In what may be a bit of an over-simplification of their approach, a height that is deemed threatening should be perceived differently than a height that is deemed not threatening. In other words, if a 10 foot cliff is deemed threatening, people should over-estimate the height irrespective of their level of arousal. On the other hand, if the 10 foot cliff is deemed non-threatening, then perception of the height should be perceived accurately (or at least not overestimated as much).

Indeed, research on motivated perception has consistently found that arousal does not underlie effects of motivation on perceptual judgments. For example, in one experiment, hungry participants judged a slice of pizza to be closer than an empty cup that was equidistant away from observers [16]. However, when the motivation to eat decreased (i.e., after satiation) the same slice of pizza was judged to be a similar distance away as the empty cup. As suggested by Balcetis, the arousal elicited by the slice of pizza should have been similar across the different motivation states, so motivation, not arousal, best predicted distance estimates to the pizza. Similar findings were obtained when the distance to an object being estimated was threatening [2]. People were exposed to a video in which a man performed either a threatening action or a disgusting action, and participants' heart rate variability, a measure of arousal, was assessed while viewing the actions. The man in the video was then brought into the lab and participants estimated the distance to him to be shorter when the man performed a threatening act compared to a disgusting act on the video. Important to note is that this effect was maintained even when arousal (measured through heart rate variability) was statistically controlled for, which suggests that arousal was not the agent of influence on perceptual estimates. Thus, arousal may be constrained by influencing distances in which there is potential for action (e.g., walking up a hill, falling off a cliff), but not perceptual estimates for non-motivationally relevant situations (e.g., seeing food when satiated, being at the base of a height) that do not require or invoke action.

Given the potential discrepancy in the literature on the role of arousal in perceptual judgments, we conducted a series of experiments to pinpoint the boundaries of an effect exploring when arousal and motivation are both manipulated, and when arousal is manipulated but the motivation to withdraw from a height is held constant. One implicit assumption we had, but never formally tested, was that standing on top of a height induces a motivation to withdraw to prevent injury. It remains unclear whether the height produces a motivational goal to withdraw or whether the experimentally induced arousal produces a motivational goal to withdraw. Thus, our first experiment in this paper examined how motivational qualities of the environment influence height estimates when people are aroused. In our second experiment, we wanted to rule out a potential confound within our induction method. The images used to induce arousal contained height-related themes. Thus, the images, and not the balcony itself, may have invoked a motivation to withdraw. The last two experiments were designed to examine whether arousal can moderate height estimates based on the intensity of the arousal and whether height estimates are changed when arousal is attributed to a source other than the height. Overall, we sought to better identify *when* and *how* arousal influences estimates of heights given the discrepancy in the literature about how arousal contributes (or not) to effects of motivation on perceptual judgments. Such an investigation is warranted because of recent concerns about the generalizability and validity of effects of emotion and motivation on perceptual judgments [17]. Determining whether these effects are limited or broad in scope will increase our understanding of the ways in which arousal and motivation are involved (or not) in everyday perceptual judgments.

### Safety of Environment

In the first experiment, we examined whether arousal influences judgments when initial appraisals of a height were either dangerous or safe. Threatening environments lead to an increased physiological response e.g., [18], [19]. In previous work, we showed that threatening environments (heights viewed from the top) were overestimated, especially when the observer was also aroused [6]. However, it is unclear whether a height that is viewed as not threatening (in other words, viewed from below) will also be overestimated when aroused. Based on a motivation account, environments perceived as threatening should elicit changes in perceptual estimates (irrespective of arousal levels), whereas those perceived as not threatening should fail to influence such estimates [15], [20]. Likewise, we also predict that the environment has to be deemed threatening in order to influence height judgments. However, contrary to the motivated perception approach, the induced arousal should activate the motivational system and increase the perceived sense of urgency resulting in a modulation of the height estimate through arousal. Thus, we investigated whether arousal influences height estimates only when a threat is present, thereby testing for whether a general perceptual bias of heights exists under conditions of arousal, or whether a motivation to withdraw (e.g., brought on by a threat) is needed for arousal to influence judgments.

### Stimulus-Driven Cues of Danger

In our previous work, we found that arousing participants by asking them to view images of height situations affected subsequent height judgments. But as mentioned above, the images themselves contained height-related themes (i.e., a skydiver, a man falling from a building) introducing a potential confound. The arousing images may have induced both a higher state of arousal and a motivation to withdraw from heights. Prior research has observed that motivations can be primed conceptually, which in turn motivates behavior that is compatible with the prime [21]. It is quite possible that the height estimates were influenced by a cognitively induced motivation to avoid a height. Therefore, we wanted to de-confound arousal and motivation to clarify the component responsible for influencing height estimates.

### Dissipation of arousal?

If arousal affects height estimates, then it should have more of an effect when feelings of arousal are intense, rather than diminished. Thus, the intensity of the arousal when making the height estimate should lead to direct changes to height estimates, irrespective of the cognitive meaning of the stimuli. This prediction would be consistent with the idea that the height triggers a motivation to withdraw, and the induced arousal signals the urgency for withdrawal. Lower urgency should correspond to a lower height estimate, whereas higher urgency should correspond to a higher height estimate. However, if motivation to act (or step away) at a height leads to height overestimation, then it should do so regardless of the level of arousal. Prior research finds that more intense levels of arousal have a greater influence on non-perceptual judgments compared to less intense levels of arousal [22]. Therefore, we sought to manipulate the intensity of the arousal at the time of the judgment to assess whether the intensity level influences height estimates.

### Attribution of arousal?

For the theories that assume that feelings can transfer from one source to another [13], [23]–[24] there is an assumption that the transferred feelings can be discounted when making a judgment. For instance, Schwarz and Clore [25] found that well-being judgments were influenced by the weather (e.g., sunny days elicited higher well-being judgments), but those biased judgments were reduced when attention was directed to the true source of the participant's feelings (i.e., the weather). This assumption is critical for examining the ability of induced feelings (potentially feelings of arousal) to influence judgments. If induced arousal is a cue of urgency to avoid the height, then participants should be able to discount the false sense of urgency. This discounting should then have a direct effect on the judgment of the height. In other words, by discounting feelings of arousal, urgency should be reduced, thereby reducing the motivation to avoid the height and possibly rendering it less dangerous. Therefore, when cues that intensify the need to avoid the height are revealed to be false, can participants correctly discount those feelings of urgency leading to a reduced height overestimation?

### Overview of Current Studies

We examine whether arousal influences height estimates when the estimates are made from a threatening location (from above) or a safe location (from below). We predict that arousal will only influence height estimates from the top. Then, we investigate the effects of situation and non-situation relevant arousal on height estimates by altering the composition of the images used to arouse participants. We predict that situation and non-situation relevant arousal will have similar effects when estimating heights because the motivation to withdraw is endemic to the height and arousal serves to activate and intensify the present motivation. Next, we test for effects of arousal intensity with non-situation relevant arousal, by introducing an intermediate or a long delay between the arousal manipulation and the perceptual judgment. We expect the long, compared to intermediate, time delay to diminish the level of arousal, which in turn would decrease the activation and intensity of the motivation to withdraw. In the final experiment, we test whether attribution of the arousal to its appropriate source will diminish overestimations of height. We predict that when the arousal is appropriately attributed to its true source (i.e., the pictures) height estimates will be reduced compared to the non-attribution condition.

## Experiment 1

The first experiment examined if arousal influences height estimates when the environment is dangerous vs. safe. Viewing a height from above is a dangerous situation (i.e., falling), which should elicit an appraisal of threat and a motivation to withdraw. That cognitive appraisal may result in an assessment of current physiological cues, and these cues, we believe, will influence judgments of the height. However, viewing the height from below is safe, so that should not elicit a motivation to withdraw. As a result, feelings of arousal may not be used to make a judgment about the height. Therefore, we predict that aroused individuals who view a height from above will estimate the height as higher compared to non-aroused individuals. Also, we predict that height estimates will be similar for the aroused and non-aroused conditions when a height is viewed from below.

### Method

#### Ethics Statement

The Queens College – CUNY Institution Review Board approved the study prior to the study being conducted, and written, informed consent was obtained for every participant.

#### Participants

Sixty-eight (45 female, 23 male) undergraduate students from Queens College participated to fulfill a course requirement. All participants had normal or corrected-to-normal vision. Their mean age was 20.47 years (*SD* = 3.26).

### Stimuli and Apparatus

#### Arousal Task

Participants saw the same pictures used in Stefanucci and Storbeck [6]. The pictures were from International Affective Picture System (IAPS) [26], and arousing pictures were used because they reliably elicit emotional and physiological arousal [18], [27]. One-hundred and twenty images were selected and divided into four groups of 30 pictures. Each participant saw one set of pictures (A and B were arousing, C and D were non-arousing). All sets contained both positive and negative images. The arousing pictures contained a mixture of height relevant (e.g., looking down from a tall building, looking down from a mountain, viewing skydivers in the air) and height irrelevant (e.g., a snarling dog, a grizzly bear, an explosion, people with guns) themes (8 pictures were height related in each picture set). Pictures were presented using PowerPoint Presentation. Summary information concerning the slides and their ratings can be found in Stefanucci and Storbeck [6].

#### Perceptual Task

Participants stood on or below a balcony that measured 5 meters high, inside a building. The balcony overlooked a hallway. A large yellow disk made of core board (44 cm in diameter) marked the distance to be judged on the ground beneath the balcony or extended from the top of the railing of the balcony.

#### Arousal Manipulation Check

The manipulation check was administered after the perceptual task. The participant was asked to “describe how you felt while viewing the pictures.” They answered this question using 6-point Likert scale with 1 being “not aroused” and 6 being “very aroused.”

#### Acrophobia Questionnaire

Participants completed the Acrophobia Questionnaire (AQ) (Anxiety Subscale) [28] to measure trait-level fear associated with heights. This scale measures the degree to which a person has fear-relevant thoughts when thinking about a variety of height environments.

#### Procedure

Participants were told that the purpose of the experiment was to test their memory for the pictures. To provide a break between the learning and testing phases of the memory task, participants were asked to complete a filler task (judging the height of a balcony). The perceptual task was described as being completely separate from the memory task. Participants were randomly assigned to either the arousing or non-arousing (neutral) condition. The experimenter was unaware of participant condition because dummy codes, known only to the first author, were used for the conditions and the experimenter left the room before image presentation began. This procedure was used in all subsequent studies.

Participants were shown one set of 30 pictures, and all pictures were presented for 3 seconds with a 250 ms delay between pictures. Immediately following picture presentation, participants left the laboratory and were walked to the balcony. The laboratory was located on the third floor, and the participants were walked down to the second floor (when estimating from above) or to the first floor (when estimating from below). There was an additional delay of approximately 12 seconds for the participants assigned to the estimating from below condition due to walking down the extra flight of stairs. Participants were randomly assigned to a viewing position. The group that estimated from the top stood on the edge of the two-story balcony (with a 0.90 m high railing) with the target placed on the ground below the balcony. They estimated the height of the balcony by positioning an experimenter to be the same distance along the balcony as the *top of the railing* (to control for eye-height differences) was to the target on the ground. The group that estimated the height from below viewed the target extended out 0.75 m from the top of the railing for the balcony. They estimated the height of the balcony by positioning an experimenter to be the same distance from the *ground beneath their feet* to the target extended from the top of the balcony railing. For both conditions, the experimenter walked backward while facing the participant and waited for the participant to tell him or her to stop. (The researchers were instructed to walk backwards at a slow to medium pace, but were told to maintain the pace until the participant instructed them to stop). Participants were encouraged to look back to the target as often as they liked and to adjust the experimenter to be closer or farther until they were satisfied with their matched estimate. Allowing them to continuously view the target during the matching task helped to ensure they were not estimating the height from memory. After estimating the height, participants then estimated the size of the target. This measure served as an indirect index of perceived height given that the perceived size of an object can be influenced by the perceived distance to the object (known as the size-distance invariance hypothesis) [29]. The experimenter stood approximately 0.61 m from the participant, and held a tape measure with the marked side facing the experimenter. The participant was informed to estimate the size of the target by treating one end of the target as one end of the tape measure and the other end of the target as the other end of the tape measure. The experimenter then pulled the tape measure out slowly until the participant believed that the length of the tape measure matched the diameter of the target. The participants were encouraged to adjust the length of the tape measure as much as they wanted in order to be as accurate as possible.

Participants were then brought back into the laboratory where they completed the Arousal Manipulation Check, the Acrophobia Questionnaire (AQ), and a demographic questionnaire. Finally, participants were debriefed and were asked specific questions to determine whether or not they linked the picture viewing to the height task. None of the participants were aware of the connection between the emotion induction and the height estimates.

### Results

For all of the reported experiments, we assessed whether the research assistant (RA) influenced height estimates by running a multivariate ANOVA. RA was entered as an independent variable along with the other manipulated variables specific to each experiment and the dependent variables were height and size estimates. All main effects involving RA and all interaction terms were non-significant, all F's<1.

#### Manipulation Check and Acrophobia Questionnaire

We assessed whether the arousal induction was successful by running a 2(Viewing Position: Top or Bottom) by 2(Arousal Level: Aroused or Neutral) factorial ANOVA on participant's self-reported level of arousal while viewing the pictures. As expected, the main effect for Arousal was significant, *F*(1, 63) = 27.38, *p*<0.01, η^2^ = 0.30, in that the arousal condition experienced higher levels of arousal during picture viewing compared to the neutral condition. The main effect for Viewing Position, *F*(1, 63) = 1.88, *p* = 0.18, η^2^ = 0.03, and the interaction between viewing position and arousal level, *F*<1, were non-significant. See Table 1 for Manipulation Check means for all experiments.

**Table 1:** Mean Arousal Ratings for the Arousal Induction for each Experiment.

	Questionnaire Types	
Conditions	Arousal	AQ
**Experiment 1**		
Arousal, From Top	3.64 (0.84)	60.93 (21.52)
Non-Arousal, From Top	2.53 (1.30)	63.53 (16.91)
Arousal, From Bottom	4.26 (1.15)	59.95 (21.77)
Non-Arousal, From Bottom	2.63 (0.90)	64.11 (25.76)
**Experiment 2**		
Arousal, Height-Relevant	4.00 (0.79)	55.53 (20.78)
Arousal, Height-Irrelevant	3.67 (1.14)	51.50 (11.90)
Neutral	2.65 (1.32)	52.12 (19.01)
**Experiment 3**		
Arousal No Delay	3.48 (0.97)	56.71 (21.00)
Arousal Delay	3.75 (0.81)	62.19 (19.59)
Neutral No Delay	2.65 (1.19)	54.92 (22.17)
Neutral Delay	2.52 (0.93)	62.21 (16.73)
**Experiment 4**		
Height-Relevant, Attribution	3.73 (1.49)	62.00 (25.16)
Height-Irrelevant, Attribution	3.27 (0.90)	51.64 (17.19)
Height-Relevant, No Attribution	4.00 (1.54)	53.75 (21.04)
Height-Irrelevant, No Attribution	3.55 (0.69)	59.27 (17.78)

*Note*. The table presents the means and standard deviations (in parentheses) for self-reported arousal on the manipulation check (Arousal) and anxiety questionnaire (AQ).

To assess whether there were group differences in trait-level fear associated with heights (AQ), we ran a 2(Viewing Position: Top or Bottom)×2(Arousal Level: Aroused or Neutral) factorial ANOVA on the total trait-level fear score. No differences were observed for self-reported trait fear of heights by condition, *F*<1.

#### Perceptual Estimates

**Figure 1:**
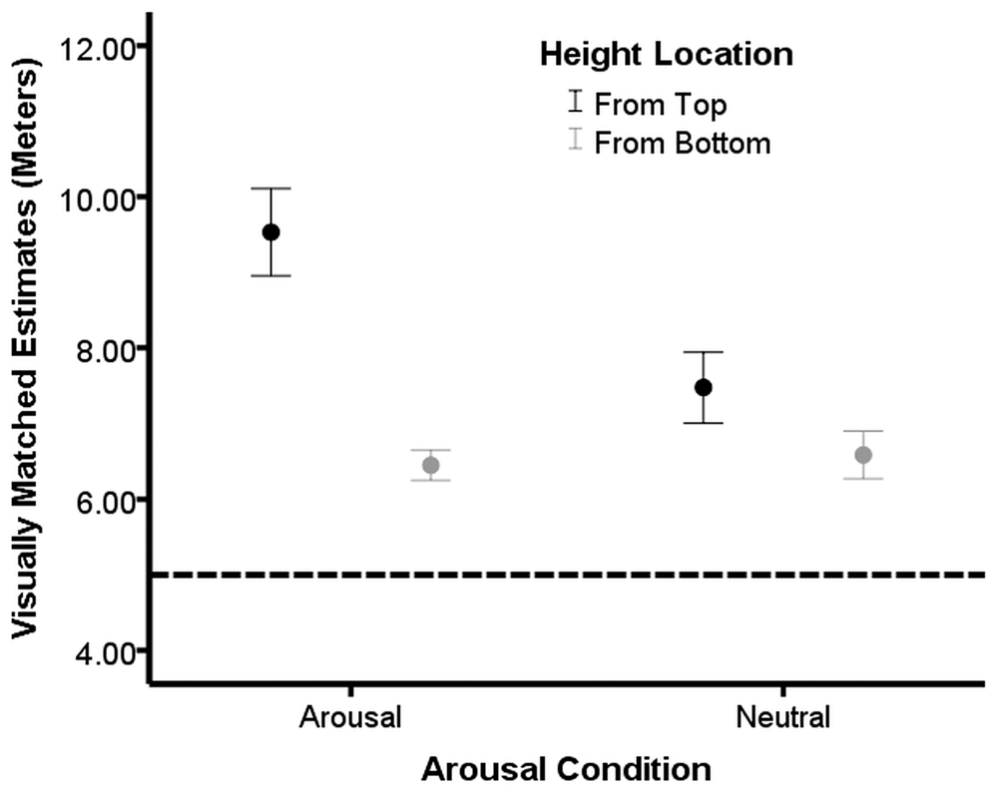
Motivation of the height interacts with arousal to moderate height estimates. Mean height estimates for the Arousal by Height from top and bottom and Non-Arousal by Height from top and bottom in Experiment 1. Bars represent one standard error of the mean and the dotted horizontal line represents the actual height of the balcony.

To examine whether location of the observer and arousal level affected height estimates, we ran a 2(Viewing Position: Top or Bottom) by 2(Arousal Level: Aroused or Neutral) factorial ANCOVA with height estimates as the dependent variable and AQ as the covariate. There were significant main effects of Viewing Position, *F*(1, 62) = 24.41, *p*<0.01, η^2^ = 0.28, and Arousal Level, *F*(1, 62) = 5.65, *p* = 0.02, η^2^ = 0.08. The covariate, AQ, was not significant, *F*<1. Estimating the balcony from the top led to greater height estimates as compared to estimates made when viewing the height from below, replicating prior work on height overestimation and viewing position [30]–[31]. Arousal level also affected overall estimation of height such that exposure to the arousal images resulted in greater height estimates when compared to viewing neutral images. However, both of the main effects were qualified by the significant interaction effect between Viewing Position and Arousal Level, *F*(1, 62) = 7.46, *p* = 0.01, η^2^ = 0.11. The interaction revealed that the high-arousal from above condition provided the tallest height estimate when compared to the other three conditions (all *p*s<0.01). We also observed that the neutral from top condition provided a higher height estimate compared to the high-arousal from bottom condition, *t*(32) = 2.17, *p* = 0.04. See Figure 1 for a graphical representation of the means.

#### Size Estimates

Another 2(Viewing Position: Top or Bottom) by 2(Arousal Level: Aroused or Neutral) factorial ANCOVA was run to analyze the effects of viewing position and arousal level on estimates of the size of the target situated at either the top or bottom of the height with AQ serving as the covariate. In contrast to the results obtained for height estimates, there was no main effect of Viewing Position, *F*(1, 62) = 1.33, *p* = 0.25, η^2^ = 0.02, Arousal Level, *F*(1, 62) = 2.17, *p* = 0.15, η^2^ = 0.03, or AQ, *F*<1, on estimates of the size of the target. The interaction between Viewing Position and Arousal Level, *F*<1, was non-significant. See Table 2 for size estimates for all experiments.

**Table 2:** Mean Size Estimates (cm) for each Experiment.

Conditions	Size Estimate
**Experiment 1**	
Arousal, From Top	43.16 (14.21)
Non-Arousal, From Top	37.25 (12.12)
Arousal, From Bottom	44.68 (10.36)
Non-Arousal, From Bottom	42.50 (9.09)
**Experiment 2**	
Arousal, Height-Relevant	45.63 (13.30)
Arousal, Height-Irrelevant	43.73 (8.00)
Neutral	36.48 (11.80)
**Experiment 3**	
Arousal No Delay	44.85 (5.56)
Arousal Delay	39.23 (8.95)
Neutral No Delay	41.53 (6.67)
Neutral Delay	40.17 (9.15)
**Experiment 4**	
Height-Relevant, Attribution	44.88 (10.18)
Height-Irrelevant, Attribution	40.76 (10.54)
Height-Relevant, No Attribution	43.42 (11.51)
Height-Irrelevant, No Attribution	47.44 (13.59)

*Note*. The table presents the means and standard deviations (in parentheses) for size estimates.

#### Correlations between Height and Size Estimates

Though size estimates did not vary based on our manipulations, we did observe a significant positive correlation between the height and size estimates, *r*(68) = 0.30, *p* = 0.01, such that greater estimates of the height were associated with larger estimates of target size. This correlation was mostly driven by significant positive correlations observed between height and size estimates when viewing from the top in either the arousal condition, *r*(15) = 0.67, *p* = 0.01, or the neutral condition, *r*(15) = 0.56, *p* = 0.03. When participants estimated the height from below, there were no significant correlations between height and size estimates in either the arousal condition, *r*(19) = −0.27, *p* = 0.27, or the neutral condition, *r*(19) = 0.23, *p* = 0.34.

### Discussion

The results of this experiment replicate those of Stefanucci and Storbeck [6]. When participants viewed the height from above and were aroused, they overestimated height. However, the current study extended the previous findings by showing that participants who viewed the height from below and were aroused did not overestimate height as compared to a non-aroused group. The results for viewing from below would be consistent with the notion that when a motivational tendency to withdraw or threat of falling is lacking, arousal fails to influence perceptual estimates [2], [16]. Given we did not find differences in estimates of height from below associated with arousal condition, we conclude that arousal may only have an influence on height judgments when the environment is appraised as threatening.

## Experiment 2

In Experiment 2, we examined whether viewing arousing images that contained height relevant themes, as used in previous experiments [6] (Exps. 1 & 2), primed danger or a motivation to avoid heights. If the pictures primed danger, it is possible that they resulted in a cognitive bias to estimate the height as taller or more dangerous leading to an exaggerated judgment rather than a change in actual perception of the height. If this is true, then height judgments should only be influenced by height-relevant, but not height-irrelevant arousal. In other words, we believe that the arousal does not have to invoke a threat of heights to influence height estimates. Rather, we suspect the height itself provides a threatening motivation (as observed in Experiment 1), which results in assessing internal arousal cues to estimate the height. Therefore, we predict that both height-relevant and height-irrelevant residual arousal should influence height estimates. This prediction is based on findings in which residual arousal not produced by the target of a judgment still influenced the judgment of that target [22], [24], [32].

### Method

#### Ethics Statement

The Queens College – CUNY Institution Review Board approved the study prior to the study being conducted, and written, informed consent was obtained for every participant.

#### Participants

Fifty-three (31 female, 21 male, 1 unreported) undergraduate students from Queens College participated in the study for course credit. All participants had normal or corrected-to-normal vision. Mean age was 21.52 years (*SD* = 5.78).

#### Stimuli and Apparatus

To find a set of pictures that were arousing with height-relevant themes (e.g., skydivers, views from tops of tall objects), we created a set of 80 pictures that included some IAPS images (all resized to the standard format of the IAPS pictures).The set of pictures that were arousing with height-irrelevant themes (e.g., snakes, guns, explosions) and the neutral (e.g., a desk, a fork, abstract art) images were obtained solely from the IAPS. We asked 43 participants (who did not estimate height) to evaluate the 80 height-related pictures on both arousal and valence dimensions using the Self-Assessment Manikin (SAM) [33] scale, which was used by Lang and colleagues to obtain normative ratings for all pictures contained in the IAPS [26]. The SAM scale consists of measures assessing two independent dimensions (1) arousal and (2) valence, each represented on a 9 point scale. For the arousal scale, one endpoint has a manikin that is jittery and the other end point has a manikin with a sleepy look. For the valence scale, one endpoint has a manikin with a smiling expression and on the other endpoint is a manikin with a frowning expression. Participants were instructed to view each picture for 5 seconds and then evaluate it on both dimensions of arousal and valence. Participants were instructed to evaluate the picture by pressing the numeric key that corresponded to the manikin that best represented how they felt. Because we were most concerned with arousal, the arousal SAM was always presented first, followed by the valence SAM. A 1 second delay occurred between trials.

Based on the ratings provided, we selected 30 of the top rated arousing pictures (e.g., individuals jumping from high heights, views from the top of buildings, bridges, and mountains, etc). We then compared the ratings for these images to the 30 pictures that were height-irrelevant and arousing from the IAPS and the 30 pictures that were height-irrelevant and non-arousing (i.e., neutral condition) from the IAPS. The three picture conditions were subjected to a one-way ANOVA with arousal as the dependent measure. As expected, the effect was significant, *F*(2, 89) = 170.00, *p*<0.01, η^2^ = 0.80. Post-hoc analyses using Tukey's HSD revealed that the two arousal picture sets did not differ, *p* = 0.99, but the height-relevant set differed from the neutral set, *p*<0.01, and the height-irrelevant set also differed from the neutral set, *p*<0.01. Another one-way ANOVA was run to compare valence ratings by condition. As expected, the main effect was significant, *F*(2, 89) = 4.64, *p* = 0.01, η^2^ = 0.10. Post-hoc analyses using Tukey's HSD revealed that the height-relevant set did not differ from the height-irrelevant set, *p* = 0.95; however, the neutral set was rated as more positive compared to both the height-relevant set, *p* = 0.04, and the height-irrelevant set, *p* = 0.02. We were not concerned that the neutral condition was rated as more positive compared to the other two conditions given our previous work found valence has little influence on the overestimation of height when aroused [6].

#### Procedure

The same cover story was used as in Experiment 1, in that participants were told that the pictures were part of a memory test and the height task served as a filler task. Participants were randomly assigned to condition. All participants viewed the height from above and estimated height as in Experiment 1.

### Results

#### Manipulation Check and Acrophobia Questionnaire

We first assessed participants self-reported level of arousal experienced while viewing the pictures (one individual failed to provide an arousing rating, but he/she was kept for height and size analyses). We ran a one-way ANOVA to evaluate self-reported level of arousal by condition, and the effect of condition was significant, *F*(2, 49) = 6.93, *p*<0.01, η^2^ = 0.22. Post-hoc analyses confirmed that the two arousal conditions (height-relevant and height-irrelevant) were not different in levels of arousal experience, *p* = 0.38. However, the arousal, height-relevant condition reported higher levels of arousal compared to the neutral condition, *p*<0.01, and the arousal, height-irrelevant condition also reported higher levels of arousal compared to the neutral condition, *p* = 0.09, although it was only marginally significant.

A one-way ANOVA was run to evaluate whether there were mean differences in trait-level fear associated with heights (AQ) among the conditions, and the condition main effect was non-significant, *F*<1. Therefore, no differences were observed among the conditions in trait-level fear associated with heights.

#### Perceptual Estimates

**Figure 2:**
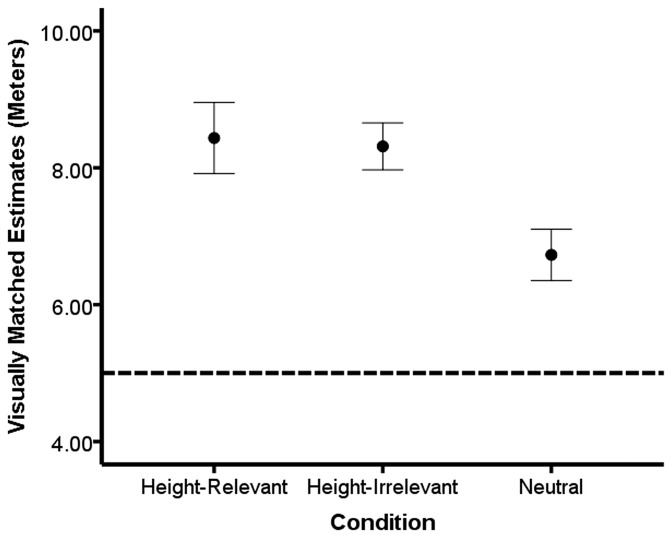
Arousal, but not motivation relevance, of the induction moderates height estimates. Mean height estimates for the height-relevant, height-irrelevant, and control conditions in Experiment 2. Bars represent one standard error of the mean and the dotted horizontal line represents the actual height of the balcony.

To examine whether height-irrelevant arousal moderates the height overestimation effect, a one-way ANCOVA was run with AQ as the covariate. As expected, a significant main effect of condition was observed, *F*(2, 48) = 4.60, *p* = 0.02, η^2^ = 0.16. The main effect for AQ was not significant, *F*<1. Post-hoc analyses using Tukey's HSD found that the height-relevant condition made similar height estimates to the height-irrelevant condition, *p* = 0.98. However, both the height-relevant, *p* = 0.02, and the height-irrelevant, *p* = 0.03, conditions gave significantly taller estimates of the balcony compared to the neutral condition (see Figure 2).

#### Size Estimates

To examine whether height-irrelevant arousal moderated estimates of the size of the target, we ran a one-way ANCOVA with AQ as the covariate. A significant main effect of condition was observed, *F*(2, 48) = 3.70, *p* = 0.03, η^2^ = 0.13. The main effect for AQ was not significant, *F*<1. Post-hoc analyses revealed that the height-relevant and the height-irrelevant did not differ with respect to their size estimates, *p* = 0.43. However, the neutral condition provided smaller size estimates compared to both the height-relevant, *p* = 0.01, and the height-irrelevant, *p* = 0.05, conditions.

#### Correlations between Height and Size Estimates

We assessed whether there was a relationship between the height and size estimates across conditions. Overall, we observed a significant positive correlation between height and size estimates, *r*(53) = 0.54, *p*<0.01, such that greater estimates of height corresponded to larger target estimates. More specifically, we found that the height-relevant condition showed this positive correlation, *r*(18) = 0.63, *p*<0.01, but both the height-irrelevant, *r*(18) = 0.35, *p* = 0.16, and the neutral, *r*(17) = 0.27, *p* = 0.29, conditions did not.

### Discussion

We found that arousal, regardless of its relevance to heights, was sufficient to produce an overestimation of height compared to the neutral condition. Moreover, we observed the same pattern of results for the size estimate. This finding suggests that the motivational relevance (i.e., height-related, arousing images) of the induction does not impact how the height is estimated. Rather, the presence or absence of an induced arousal state was the best predictor of height estimates. Moreover, in our prior work [6] we observed that when the images were positive and approach-oriented they had the same influence on height estimates as negative and withdrawal-oriented images. In sum, we suggest that estimating a height from above is threatening, and that arousal cues irrespective of the theme or motivational quality serve to increase height and size estimates.

## Experiment 3

Experiment 3 was designed to examine whether the intensity of arousal also contributes to height overestimation, while holding the motivational quality of the stimuli constant (e.g., always threatening). If the arousal from the pictures is combined with arousal produced by the target, then the intensity of the induced arousal should moderate height estimates. In a previous study, we found that participants who were asked to up-regulate their emotional experience when viewing arousing pictures increased their height estimates compared to participants who simply viewed arousing images [6]. In the current study, we examine whether reducing the intensity of the arousal once it is present can eliminate the height overestimation observed after viewing arousing pictures. If we demonstrate that overestimation of height is dependent on the intensity of arousal, then this would provide strong evidence that arousal is contributing to height estimations and would also allow us to understand the time course of such an effect.

In order to manipulate the intensity of the arousal, we adopted a timing paradigm from Cantor et al. [22]. They induced arousal and participants judged erotic films immediately after viewing or after an intermediate or a long delay. Only the intermediate delay influenced judgments of erotic films. The immediate delay failed to influence judgments because participants were aware of the true source of their feelings, whereas, the long delay failed to influence judgments because the arousal wore off. Research by Cantor et al. [22] revealed that a nine-minute delay was sufficient to return physiological markers of arousal back to a baseline state, whereas within the intermediate condition (5 minutes post induction) arousal was still elevated from baseline. We predict that with an intermediate delay both height-relevant and -irrelevant arousal conditions will show increased height estimates, but after a long delay both height-relevant and -irrelevant arousal conditions will estimate height as the neutral condition would.

We manipulated timing by having participants in the intermediate delay condition estimate height following the arousal induction, whereas participants in the long delay condition completed a Big Five personality inventory between the arousal induction and the height estimate. The Big Five personality task was selected because none of the questions relate to height or to arousal. To ensure that completing the Big Five personality measure alone did not influence height we included a neutral condition in which participants viewed neutral pictures then completed the Big Five measure prior to estimating the height. As mentioned previously, Cantor et al. [22] found that a long delay (9 minutes) between arousal induction and judgment did not influence the judgments. However, the intermediate delay of 5 minutes between arousal and evaluation increased the evaluation of the erotic film. For our experiment, the induction takes place in the lab and the participants have to walk to the balcony, so there is a delay of 3–5 minutes (i.e., walk+instruction) when no additional time is added. Therefore, we ran a ∼5 minute delay (time between induction and estimates in previous experiments) and a ∼9 minute delay condition (the Big Five personality questionnaire takes about 4–5 minutes to complete).

### Method

#### Ethics Statement

The Queens College – CUNY Institution Review Board approved the study prior to the study being conducted, and written, informed consent was obtained for every participant.

#### Participants

Ninety (59 female, 27 male, 4 non-reported) undergraduate students from Queens College participated in the study for course credit. All participants had normal or corrected-to-normal vision. Mean age was 20.93 years (*SD* = 4.43).

#### Stimuli, Apparatus, and Procedure

The two arousing conditions viewed 30 height-irrelevant, arousing pictures and the neutral conditions viewed 30 height-irrelevant, neutral pictures. The procedures were identical to the procedures in Experiment 1, except that the long delay condition completed the Big Five Inventory, which had 44 items, immediately after viewing the pictures. Once they completed the inventory they were walked to the balcony to estimate the height.

### Results

#### Manipulation Check and Acrophobia Questionnaire

A 2×2 (Arousal [arousal, neutral] x Delay [intermediate delay, long delay]) factorial ANOVA was run to evaluate whether self-reported level of arousal were influenced by the arousal and delay manipulations. The effect of arousal was significant, *F*(1, 81) = 23.10, *p*<0.01, η^2^ = 0.22. The arousal condition reported higher levels of arousal compared to the neutral condition. The main effect for delay, F<1, and the interaction effect, *F*(1, 81) = 1.24, *p* = 0.27, η^2^ = 0.02, were both non-significant.

The same 2×2 factorial ANOVA was run to evaluate whether arousal and delay interacted to influence trait-level fear of heights (AQ). The two main effects of arousal, *F*<1, and delay, *F*(1, 81) = 2.13, *p* = 0.15, η^2^ = 0.03, and the interaction of arousal and delay, *F*<1, were all found to be non-significant. Therefore, no differences were observed among the conditions in trait-level fear associated with heights.

#### Perceptual Estimates

First, to assess whether completion of the Big Five personality questionnaire influenced height estimates, we ran a 2(Arousal)×2(Delay) factorial ANCOVA with the personality items as covariates, and none of the factors achieved a level of significance, all *p*'s>0.12. Thus, we removed the Big Five Inventory from the analysis, because it had no influence on height estimates.

**Figure 3:**
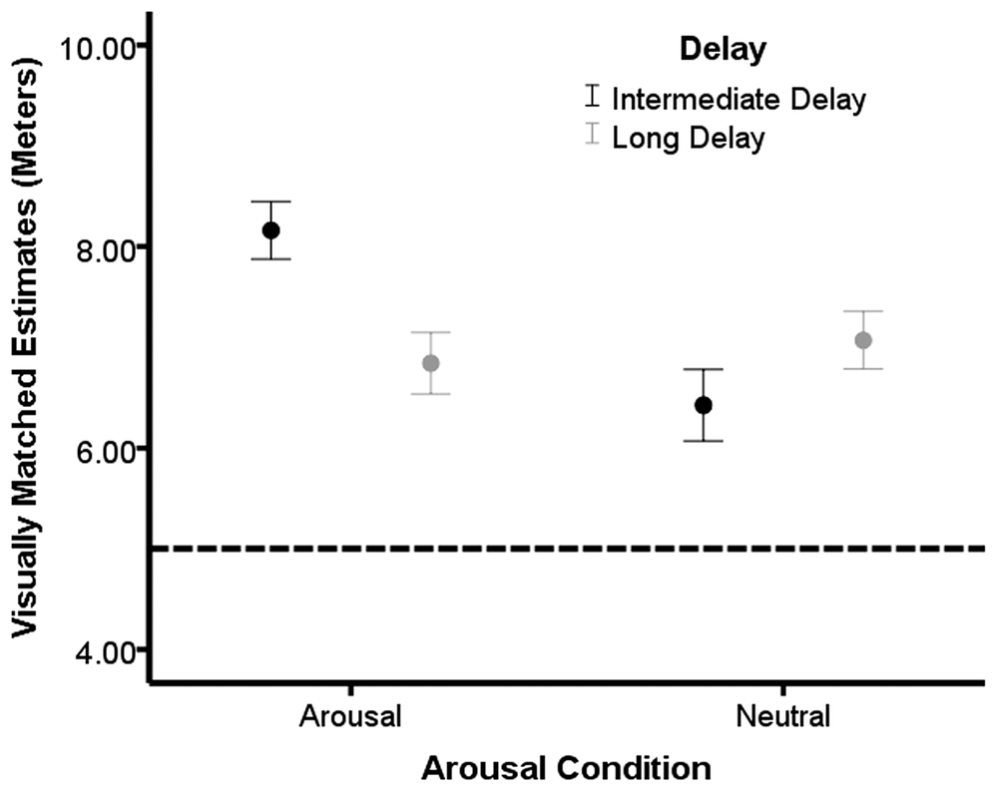
Intensity of the arousal moderates height estimates. Mean height estimates for the arousal intermediate delay, arousal long delay, and neutral delay conditions in Experiment 3. Bars represent one standard error of the mean and the dotted horizontal line represents the actual height of the balcony.

A 2×2 (Arousal [arousal, neutral]×Delay [intermediate delay, long delay]) factorial ANCOVA was run to evaluate whether a delay between the arousal induction and the estimation task influenced height estimates (AQ served as the covariate). We observed a significant effect for Arousal, *F*(1, 81) = 7.06, *p* = 0.01, η^2^ = 0.12; however, this main effect was qualified by the predicted Arousal by Delay interaction, *F*(1, 81) = 9.46, *p*<0.01, η^2^ = 0.11. The main effect for Delay was not significant, *F*<1. We also observed a significant effect of the covariate AQ, *F*(1, 81) = 10.93, *p*<0.01, η^2^ = 0.12. By examining the interaction shown in Figure 3, as predicted, the intermediate arousal condition provided the highest height estimates compared to the other three conditions (all *p*s<0.05).

#### Size Estimates

Another 2×2 (Arousal×Delay) factorial ANCOVA was run to determine whether a delay between the arousal induction and the estimation tasks influenced size estimates of the target (AQ served as the covariate). A trend was observed for Delay, *F*(1, 81) = 2.70, *p* = 0.10, η^2^ = 0.03, such that the intermediate delay condition had larger size estimates. However, the main effect for Arousal, *F*<1, and AQ, *F*(1, 81) = 2.16, *p* = 0.15, η^2^ = 0.03, and the Arousal by Delay interaction, *F*(1, 81) = 1.93, *p* = 0.17, η^2^ = 0.02 were all non-significant.

#### Correlations between Height and Size Estimates

We also assessed the correlation between the height and size estimates. When all conditions were assessed, there was a positive correlation between height and size estimates, *r*(89) = 0.55, *p*<0.01, such that greater estimates of height corresponded to larger estimates of target size. This significant positive correlation was present for the Arousal, Long Delay, *r*(21) = 0.48, *p* = 0.03, the Arousal, Intermediate Delay, *r*(22) = 0.56, *p* = 0.01, and the Neutral, Intermediate Delay, *r*(23) = 0.77, *p*<0.01, conditions. For the Neutral, Long Delay condition, there was a trend toward a positive correlation, *r*(23) = 0.38, *p* = 0.07.

### Discussion

Experiment 3 demonstrated that when arousal dissipates, an overestimation of height compared to the neutral condition does not occur. In addition, when arousal has not dissipated, overestimation of height compared to the long delay and neutral conditions does occur, replicating the findings of the previous experiments. Taken together, these results suggest that arousal may serve as an urgency cue for how threatening a height is estimated to be. When arousal cues are present, even when from an unrelated source, it may elicit a greater sense of urgency to withdraw from the height, which results in a higher overestimation.

## Experiment 4

We assume that feelings of arousal, activated when viewing the images in the previous experiments, are attributed to the height resulting in an overestimation [6], [13]. Thus, increases in overestimation of height may be dependent upon the ability of the arousal from an alternate source to be misinterpreted as stemming from the height. We do note that in our prior research almost all participants failed to realize the connection between the arousal induction and the height estimate [6]. However, we have yet to experimentally test whether arousal from the pictures is being attributed to the balcony height (i.e., a dangerous environment). Moreover, if people can become aware of how the irrelevant source is influencing their feelings while on the balcony, can those feelings be adjusted for in order to make a less biased estimate of the height?

Prior research has tested attribution by drawing attention to the true source of the emotional feelings. When participants attribute their feelings to the irrelevant source, subsequent judgments are adjusted to discount the emotional feelings from that source [22], [25], [34]. In this experiment, we examine whether perceptual estimates are moderated when the true source of the feelings are made salient, to test whether height overestimations may result, in part, from a misattribution of arousal. We hypothesized that when participants attribute their feelings to the arousal manipulation (i.e., the pictures), their perceptual estimates will be reduced because arousal will no longer be used as information for the height judgment.

### Method

#### Ethics Statement

The Queens College – CUNY Institution Review Board approved the study prior to the study being conducted, and written, informed consent was obtained for every participant.

#### Participants

Forty-five (23 female, 22 male) undergraduate students from Queens College participated to fulfill a course requirement. All participants had normal or corrected-to-normal vision. Mean age was 21.13 years (*SD* = 5.38).

#### Stimuli and Apparatus

The stimuli and materials were identical to those in Experiment 2, with the exception of the attribution questions. In the attribution condition, participants were asked three questions designed to draw attention to the feelings of arousal elicited by the pictures (see Appendix S1) [35]. For the non-attribution condition, the participants viewed the arousing images and provided estimates of the height.

#### Procedure

Four conditions were run in total: a height-relevant arousing attribution condition, a height-irrelevant arousing attribution condition, and both arousing conditions without attribution manipulations. The procedure was identical to Experiment 2, except that participants assigned to the attribution conditions were asked three questions to draw attention to the arousing nature of the pictures. The questions were asked immediately after viewing the pictures while still in the lab, and then participants walked to the balcony to complete the height estimation task.

### Results

#### Manipulation Check and Acrophobia Questionnaire

Given that the attribution manipulation was designed to make participants aware of the source of the arousal feelings, we anticipated that all conditions should experience a similar level of arousal when viewing the pictures. To assess self-reported arousal, we ran a 2 Arousal Type (height-relevant vs. height-irrelevant)×2 Attribution (attribution vs. no attribution) between-participants ANOVA. No significant main effect for Arousal Type, *F*(1, 41) = 1.56, *p* = 0.22, η^2^ = 0.04, nor Attribution, *F*<1, was observed, and there was no significant interaction between Arousal Type and Attribution, *F*<1. Thus, all four conditions experienced a similar level of arousal while viewing the pictures.

To assess whether there were group differences in self-reported trait-level fear associated with heights (AQ), we ran a 2 Arousal Type×2 Attribution between-participants ANOVA on mean trait-level fear of heights. The main effects for Arousal Type, *F*<1, and Attribution, *F*<1, were both non-significant. The interaction of Arousal Type and Attribution was also non-significant, *F*(1, 41) = 1.68, *p* = 0.20, η^2^ = 0.04. Therefore, there were no group differences for self-reported, trait-level fear of heights.

#### Perceptual Estimates

**Figure 4:**
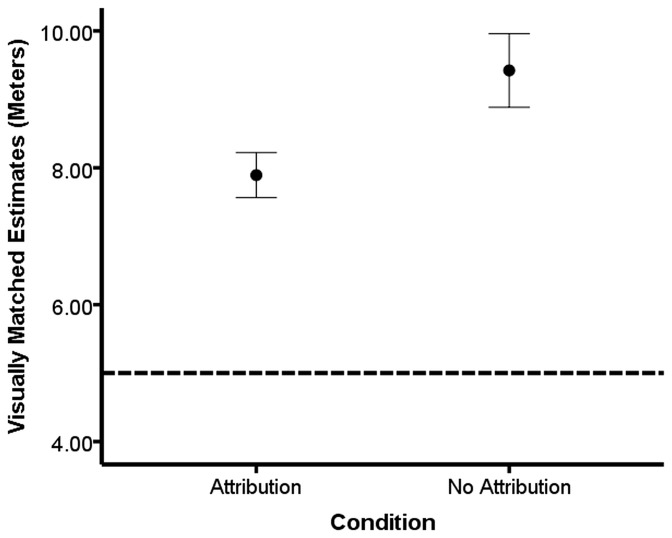
Attribution of arousal reduces height estimates. Mean height estimates for the attribution and non-attribution conditions (collapsed across image themes) in Experiment 4. Bars represent one standard error of the mean and the dotted horizontal line represents the actual height of the balcony.

To determine whether attributions made about arousing pictures would influence height estimates, we ran a 2 Arousal Type (height-relevant vs. -irrelevant pictures)×2 Attribution (attribution vs. no attribution) between-participants ANCOVA with height estimates as the dependent variable and AQ as the covariate. As expected, we observed a significant main effect for Attribution, *F*(1, 40) = 5.46, *p*<0.03, η^2^ = 0.12, but there was no main effect of Arousal Type, *F*<1, and AQ, *F*<1, nor an interaction between Arousal Type and Attribution, *F*<1 (See Figure 4). The main effect of Attribution revealed that the non-attribution conditions estimated the height to be taller than the attribution conditions.

#### Size Estimates

The same 2(Arousal Type) by 2(Attribution) between-participants ANCOVA used to analyze height estimates was re-run with size estimates as the dependent measure and AQ as the covariate. The main effects for Arousal, *F*<1, Attribution, *F*<1, and AQ, *F*(1, 40) = 5.46, *p* = 0.11, η^2^ = 0.07, and the Arousal by Attribution interaction, *F* = 1, were all non-significant.

#### Correlations between Height and Size Estimates

We assessed the correlation between height estimates and size estimates and found a significant positive correlation, *r*(45) = 0.33, *p* = 0.03, such that greater height estimates corresponded to larger estimates of target size. Given that each group had fewer than fourteen people, we collapsed across the Attribution conditions to examine correlations between height and size estimates. The positive correlation remained significant for both the Attribution, *r*(22) = 0.44, *p* = 0.04, and the no Attribution, *r*(23) = 0.43, *p* = 0.04, conditions.

### Discussion

When arousal was appropriately attributed to the images, participants' judgments of the height were reduced. This finding suggests that feelings of arousal produced by an irrelevant source can be used as cues when judging heights, but may not always be. When the source of the arousal is correctly attributed to the arousal induction, participants are able to discount those feelings as information for height estimates. Also, when the feelings were discounted, we believe that motivation to avoid the height may have still been present, but less urgent, which translated into a reduction in overestimation of the height.

## General Discussion

These experiments explored how the presence and intensity of arousal interacted with motivation to influence how people judge the extent of a height. We observed that arousal influences estimates of height when the height is viewed from the top, but not from below, suggesting that an appraisal of danger is necessary for arousal to serve as information when making judgments of height. This result also suggests that when motivation to withdraw is not as high (because one is standing on the ground) overestimation may dissipate. Moreover, we found that viewing either height-relevant or -irrelevant arousing pictures produced height overestimation. This suggests that arousal does not have to be specific to the situation in order to influence perceptual judgments. However, increasing the delay between the arousal manipulation and the height estimate resulted in a reduction in height overestimation, suggesting that the intensity of arousal needs to be sufficient for overestimation to occur. Finally, we found that by directing attention to the feelings elicited by the arousal manipulation, participants discounted those feelings from the pictures when making a height judgment, again resulting in decreased overestimations of height. The reduction of height overestimation through a correct attribution of the source of the arousal, suggests that arousal is being misattributed to influence judgments.

Our work involved making perceptual judgments, which adds to the previous literature claiming that non-specific sources of arousal influence cognitive judgments. For example, prior work found that exercise induced arousal was misattributed to an erotic film and influenced judgments of excitement about the film [22]. Research examining long-term memory has also found that non-specific sources of arousal enhance memory consolidation. For instance, arousal inductions using a cold pressor task [36] or a Trier social stressor task [37] both resulted in enhanced long-term memory of emotional stimuli. Our results are also theoretically consistent with the theory of excitation transfer proposed by Zillmann [24], which argued that non-specific cues of arousal can be transferred from one source to another to influence behavior. Adding to this literature, we found that even height estimates are subject to the influence of non-specific arousal cues when the extent is appraised as dangerous.

Although feelings of arousal from multiple sources can influence perceptual and cognitive judgments, the timing of that influence is important. There exists a critical period in which the irrelevant source of arousal and the target source of arousal need to co-occur. A long delay between the arousal induction and the judgment can lead to a dissipation of aroused feelings, thereby reducing or nullifying the effect of residual arousal on judgments. For example, Dutton and Aaron [38] had male participants walk over a rickety bridge and either immediately talk with an attractive female experimenter or the participants “walked off” the arousal feelings elicited when crossing the rickety bridge and then they spoke with the female experimenter. The participants who did not “walk-off” the arousal were more likely to attribute the aroused feelings to the experimenter resulting in more participants phoning her later. Our findings similarly suggest that concurrent or recent feelings of residual arousal will be most likely to influence perceptual judgments.

In addition to the dissipation of feelings due to a delay before making a judgment, feelings that have been attributed to their true source fail to influence height judgments. These results are consistent with the arousal-as-information approach [13], see also [24], [39] for a similar theoretical view. The main tenet of the affective arousal-as-information approach is that embodied cues of valence and arousal provide signals of value (e.g., good, bad) and importance, respectively. However, when the feelings are appropriately attributed to their true source, effects of emotion on judgments are either nullified or in some cases reversed when compared to non-attribution conditions [23], [34]. Similar findings were observed in the current experiment. When individuals were able to correctly attribute the source of the arousal cues to the arousal induction, those feelings were not used as information about the height judgment. Rather those feelings were discounted at the time of judgment, and consequently, height estimates were lower than estimates provided by individuals in the non-attribution conditions.

### How does arousal influence perception?

Within our current studies, we also observed that even when there was potential for motivated action (withdrawal), arousal could weakly or strongly intensify the height overestimation effect. In other words, once a situation is deemed dangerous and produces arousal, a withdrawal motivation may result that intensifies the feelings of urgency to act. However, it is unclear whether there is a direct link between arousal and changes in perception. One possibility is that arousal serves to narrow attention. There is consistent evidence suggesting such an effect exists [40]–[42]. However, the narrowing of attention could focus the participant on internal, embodied cues, or external, perceptual cues in the environment, or both. That is, if the event is appraised as threatening, it should increase physiological arousal. Indeed, prior research often finds that emotional stimuli become more conscious and the foci of attention during the appraisal process [43], [44]. Thus, attention may shine a spotlight on the embodied cues intensifying the feelings of danger, leading to an overestimation of height in our experiments.

In contrast, arousal may also narrow attention toward the perceptual environment. The arousal biased competition theory has proposed that arousal will increase attention to the most salient cue and reduce processing of non-salient cues [45]. Research by Gable and Harmon-Jones [7] also finds that when people are in approach or withdrawal states, increases in intensity of the motivational state serve to narrow visual attention. When estimating height, multiple cues are available and used to estimate those extents. A narrowing of attention to one aspect of the environment (i.e., how tall the railing is for safety purposes) could reduce the number of perceptual cues available to judge the extent, thereby fostering a greater reliance on non-visual cues such as arousal. Future studies could experimentally test these ideas by manipulating the availability of visual cues within the environment, possibly using virtual reality, such that the available visual cues could specifically aid or worsen height judgments. The reliance on arousal could then be better assessed in perceptual environments with many and few visual cues.

### Does arousal influence perception or judgments?

To understand observers' perception of the environment, we must ask them about what they see. Thus, both a perceptual representation of the environment (in this case, a height) and a decision process are required in order to form an estimate of the environment. These decision processes could be influenced by arousal, or the perceptual representation itself could be altered. If arousal acts on the perceptual representation itself, then we could argue that participants are *seeing* the height as taller when they are aroused. Alternatively, arousal could act on the decision processes, which are required to construct a response resulting in an effect on judgments that are made after evaluation of the representation has occurred. The behavioral data collected in these experiments does not allow us to definitively claim that *perception* of the height was altered by arousal, but we are sure that arousal influenced either the representation or the decision process given our reliable and consistent effects. We used two estimates of heights, a perceptual matching task and a size estimate of the target. From a participant's perspective, the matching task is more susceptible to cognitive bias than the size estimates because it is more difficult to discern how to bias size estimates to be consistent with the hypothesis that arousal should influence height (i.e., if one believes they should say the height is taller, then size-distance invariance states they should also say the target is larger [29]). Therefore, we might suspect that the size estimates would be less susceptible to cognitive biases. When examining the size estimates, we observed mixed results. For Experiments 1 and 4, we failed to find differences with respect to size estimates. For Experiments 2 and 3, we observed differences in size estimates that mimicked the findings of the matching task. The reason for these mixed patterns of results remains unclear, as there could be several possibilities for the differences. Also, there may be factors that moderate or mediate whether height estimates are driven by changes in perception or cognition. However, other work has shown that spatial perception and contrast sensitivity may be affected by arousal [4], [14], [46]. In conjunction with our findings, the literature suggests that the effect of arousal on perception (whether it is the representation or the decision process) generalizes across stimuli in different domains of perceptual study.

### Was arousal manipulated?

One limitation of this research was our inability to associate experienced arousal with height estimates. We did not do this because of the design of the experiment. Arousal was assessed after the height judgment, which introduces a time delay between viewing the pictures and completing the manipulation check reducing the reliability of the manipulation check ratings. Although there seems to be a simple remedy to fix this issue – assessing feelings of arousal after viewing the pictures – this design has its limitations as well. As evident in Experiment 4, observers' height estimates were influenced when they correctly attributed their residual arousal to the induction. So, assessing feelings of arousal earlier could contaminate height judgments. Future research could assess manipulated levels of arousal and their influence on height estimates with physiological measurements related to heart-rate variability and galvanic skin response (GSR). However, we do note that obtaining physiological correlates of arousal is tenuous and often not reliable.

### What is the role of motivation?

In our set of experiments, we must consider that there were two situations in which motivation and arousal were present; 1) the induction, and 2) the height. For the induction, the current findings and the findings presented in Stefanucci and Storbeck [6] suggest that the motivation and/or valence of the induction does not impact height estimates to the degree that arousal does. Across the studies presented here, as well as those in Stefanucci and Storbeck [6], images were manipulated to be exciting (approach-oriented), fearful (avoidance-oriented), height-relevant (avoidance of heights) or height-irrelevant (non-specific avoidance-orientation). The motivation orientation of the images did not have an influence on perception to a greater degree than the level of arousal. As for the height, we did observe that a motivation to withdraw (looking down vs. looking up) is necessary to produce an overestimation effect. However, level of arousal still moderated the extent of the overestimation, with high levels of arousal increasing the overestimation compared to low levels of arousal. Thus, we suggest that motivation is directly relevant to the situation being judged, and that the motivation-orientation of the arousal induction does not influence the perceptual estimate.

The motivated perception approach suggests that motivation may change the perception of distances [15]. Specifically, objects that are desirable (approachable) or threatening (avoidable) are perceived to be closer in proximity than non-desirable or non-threatening objects. Moreover, the approach also suggests that arousal does not underlie these changes in perceived proximity. With respect to our findings, we do suggest that the location of the judgment does need to be threatening to elicit changes in perception (see Exp. 1), which would be consistent with the motivated perception approach. However, our results argue against the direction of change in proximity (we find threatening heights are perceived as farther rather than closer) and suggest a role for arousal in perception. Namely, when heights are to be avoided, people tend to overestimate the height. And this holds true for people not induced into an aroused state [31], for those who have a fear of heights [47]–[48], and for people induced into an aroused state prior to estimating the height [6]. But, the degree of the overestimation does appear, in part, to be dependent on the level of arousal, with higher states of arousal leading to higher overestimations of the height. In sum, the findings related to arousal influencing height estimates as just described somewhat contradict the motivated perception approach in that a motivation to avoid a height makes the extent look taller, not shorter, and that arousal moderates perceptual estimates of the extent.

## Conclusions

Our results suggest that arousal may increase perceptual estimates by serving as an intensifier of the already present motivation to avoid a height (see also [5]). As observed, these arousal cues can be quite general, the cues need to be intense at the time of judgment, and the cues can be easily misattributed from an irrelevant source to the height. However, in order for the misattributed arousal to influence height judgments, the height has to be potentially dangerous. These results support previous theories suggesting that arousal can bias judgments [13], [24], [39] and also extend these findings to include perceptual judgments. In sum, the ability to transfer non-specific arousal to perceptual judgments is adaptive in that it reduces the likelihood of approaching a dangerous environment given the presence of arousal.

## Supporting Information

Appendix S1 **The directions and questions used for the attribution manipulation (Experiment 4).**
(DOCX)
