# Supplementary material for: Conditions under which Arousal Does and Does Not Elevate Height Estimates
Source: PLoS One. 2014 Apr 3;9(4):e92024. doi: 10.1371/journal.pone.0092024 (PMC3974728; doi:10.1371/journal.pone.0092024)
Supplement: Appendix S1 — The directions and questions used for the attribution manipulation (Experiment 4). (DOCX) [file pone.0092024.s001.docx]

Appendix A

For each of the statements listed below, participants responded by rating how much they endorsed that statement using the following labels:

1 = Strongly Disagree

2 = Moderately Disagree

3 = Slightly Disagree

4 = Slightly Agree

5 = Moderately Agree

6 = Strongly Agree

**Statement 1:** When I was viewing the pictures, I could detect feelings of tenseness, anxiety, and arousal.

**Statement 2**: I think that any feelings of anxiety or arousal I feel at the present moment come mainly from the pictures I just viewed.

**Statement3**: Without just viewing those pictures, I would probably not feel tense, anxious, or emotional arousal right now.
